# Genomic epidemiology and molecular characteristics of *bla*_NDM-1_-positive carbapenem-resistant *Pseudomonas aeruginosa* belonging to international high-risk clone ST773 in the Gauteng region, South Africa

**DOI:** 10.1007/s10096-024-04763-5

**Published:** 2024-01-24

**Authors:** Hyunsul Jung, Johann D. D. Pitout, Yasufumi Matsumura, Kathy-Anne Strydom, Chanel Kingsburgh, Marthie M. Ehlers, Marleen M. Kock

**Affiliations:** 1 Department of Medical Microbiology, University of Pretoria, Pretoria, South Africa; 2 Division of Microbiology, Alberta Public Laboratories, Cummings School of Medicine, University of Calgary, Calgary, Canada; 3 Department of Clinical Laboratory Medicine, Kyoto University Graduate School of Medicine, Kyoto, Japan; 4 Ampath National Reference Laboratory, Centurion, South Africa; 5 Department of Medical Microbiology, Tshwane Academic Division, National Health Laboratory Service (NHLS), Pretoria, South Africa

**Keywords:** Genomic epidemiology, Carbapenem-resistant *Pseudomonas aeruginosa*, ST773, *Bla*_NDM-1_, Integrative and conjugative element, South Africa

## Abstract

**Purpose:**

The emergence of carbapenem-resistant *P. aeruginosa* (CRPA) harbouring acquired carbapenemase genes (*bla*_VIM_, *bla*_IMP_ and *bla*_NDM_) has become a global public health threat. Three CRPA isolates included in the study had an extensively drug-resistant phenotype with susceptibility to colistin only and were positive for the *bla*_NDM-1_ gene. The current study aimed to investigate the genomic epidemiology and molecular characteristics of the *bla*_NDM-1_-positive CRPA isolates collected from the Gauteng region, South Africa.

**Methods:**

Short read whole genome sequencing (WGS) was performed to determine sequence types (STs), genetic relatedness, resistome, virulome and the genetic environment of the *bla*_NDM-1_ gene.

**Results:**

The WGS and phylogenetic analyses revealed that the study isolates belonged to an international high-risk clone ST773 and belonged to the same clade with eight *bla*_NDM-1_-positive ST773 isolates from Hungary, India, Nigeria, South Korea and USA. The study isolates harboured a wide repertoire of intrinsic and acquired antibiotic resistance genes (ARGs) related with mobile genetic elements, porins and efflux pumps, as well as virulence factor genes. The clade-specific ARGs (*bla*_NDM-1_, *flo*R2/*cml*A9, *rmt*B4, *tet*G) were found in a putative integrative and conjugative element (ICE) region similar to ICE*6660-like*.

**Conclusion:**

As ICE carrying the *bla*_NDM-1_ gene can easily spread to other *P. aeruginosa* isolates and other Gram-negative bacteria, the findings in this study highlight the need for appropriate management strategies and active surveillance of CRPA isolates in the Gauteng region, South Africa.

## Introduction

*Pseudomonas aeruginosa* is an important opportunistic pathogen causing a wide range of healthcare-associated infections, including ventilator-associated pneumonia, catheter-associated urinary tract, surgical site, burn wound and bloodstream infections (BSIs) [1–3]. In recent years, the emergence of carbapenem-resistant *P. aeruginosa* (CRPA), along with international/global “high-risk clones” (specific epidemic *P. aeruginosa* clones that exhibit multidrug-resistant (MDR) or extensively drug-resistant (XDR) phenotypes and are characterised by worldwide dissemination in the hospital environments; e.g. sequence type (ST) 235, ST111, ST233, ST308, ST654 and ST773), has become a major national and international public health concern due to its intrinsic and acquired ability to rapidly become resistant against multiple antibiotics including carbapenems [4–6].

Carbapenem resistance in *P. aeruginosa* can be achieved by one or a combination of resistance mechanisms, which can include (i) chromosomal mutations that alter the OprD porin activity (reduced permeability) and promote down-regulation (e.g. mutation in *mexT*) or loss of the OprD porin; (ii) overexpression of the efflux pump systems and chromosomal cephalosporinases (AmpC); and (iii) enzymatic inactivation of carbapenems by carbapenemases that are acquired through horizontal transfer (HGT) of mobile genetic elements (MGEs) [6–9]. The most common carbapenemases found in *P. aeruginosa* are metallo-β-lactamases (MBLs), such as the Verona integron–encoded MBL (VIM) and imipenem-hydrolysing MBL (IMP) [10–12]. In contrast, another type of MBL called the New Delhi MBL (NDM) is rare in *P. aeruginosa* (ranging from 0.04% (3/8010) to 0.88% (26/2953)) [10, 12]. Amongst the NDM variants, NDM-1 is the most widely spread and the most prevalent variant that can be found across the *Enterobacterales* order (*K. pneumoniae*, *E. coli*, the *Enterobacter cloacae* complex and others) and 10 bacterial families of *Gammaproteobacteria*, including the *Moraxellaceae* family (*Acinetobacter* spp.) and the *Pseudomonadaceae* family (*Pseudomonas* spp.) in over 80 countries on all continents except Antarctica [10, 13, 14].

In South Africa, the first laboratory-confirmed case of NDM-1 was reported in 2011 in a carbapenem-resistant *E. cloacae* isolate, which was recovered from a patient admitted to an academic hospital in Johannesburg [15]. Four years later, the *bla*_NDM-1_ gene was reported in *P. aeruginosa* isolates from cystic fibrosis patients in Durban [16]. However, the molecular epidemiological characteristics of these isolates or the genetic context of the *bla*_NDM-1_ gene in these isolates was not further explored. Here, the presence of the *bla*_NDM-1_ gene was revealed by short read whole genome sequencing (WGS) in three CRPA isolates belonging to ST773 in the Gauteng region, South Africa. This study further aimed to elucidate the genomic epidemiology and molecular characteristics of the *bla*_NDM-1_-positive ST773 CRPA isolates and the genetic environment of the *bla*_NDM-1_ gene.

## Materials and methods

### Bacterial isolates, species identification and antimicrobial susceptibility testing

Three *bla*_NDM-1_-positive *P. aeruginosa* isolates from a biobank collection of 82 clinical CRPA isolates at Department of Medical Microbiology, University of Pretoria, were investigated in this study (referred as “the study isolates” below). The biobank collection consisted of any consecutive *P. aeruginosa* isolates showing resistance to imipenem (IPM) or meropenem (MEM) during routine microbiological analysis and antimicrobial susceptibility testing (AST) by public and private diagnostic laboratories in Pretoria, South Africa, from May 2016 to September 2019. Corresponding isolate data (collection date, specimen type, hospital ward, city) of the study isolates were obtained from the diagnostic laboratories. One of the study isolates (PA-P104) was originated from a sputum specimen collected during December 2017 from a patient admitted to the general ward of a private hospital in Johannesburg, South Africa. The other two study isolates (PA-D5 and PA-A18) were originated from pus and endotracheal aspirate specimens collected during March and June 2019 from patients admitted to the intensive care unit (PA-D5) and the high care ward (PA-A18) of private hospitals in Benoni and Boksburg (situated approximately 27 km and 37 km from Johannesburg), respectively.

The routine species identification and AST were performed using the VITEK® 2 system (bioMérieux SA, Marcy l’Etoile, France) with the VITEK® GN cards (bioMérieux SA, Marcy l’Etoile, France). The tested antibiotics were piperacillin-tazobactam (TZP), ceftazidime (CAZ), cefepime (FEP), imipenem (IPM), meropenem (MEM), amikacin (AMK), gentamicin (GEN) and ciprofloxacin (CIP). An additional disc diffusion assay was performed to confirm IPM resistance for isolate PA-D5, as the VITEK minimum inhibitory concentration (MIC) was not available. Colistin (CST) MIC was determined using the broth microdilution (BMD) method. The MICs and zone diameter interpretations were as per the European Committee on Antimicrobial Susceptibility Testing (EUCAST) Clinical Breakpoints Table version 9.0 [17]. The *P. aeruginosa* isolates were defined as MDR if isolates were resistant to three or more tested antibiotic classes, or as XDR if isolates were resistant to all but two or less antibiotic classes, as defined by Magiorakos et al. [18]. The study was approved by the Faculty of Health Sciences Research Ethics Committee, University of Pretoria (ethics reference no. 671/2018).

### Whole genome sequencing and bioinformatics analyses

Genomic DNA was extracted using the MasterPure™ Complete DNA and RNA Purification Kit (Lucigen Corporation, Middleton, WI, USA) as per manufacturer’s instructions. Libraries were prepared by using the Riptide™ High-Throughput Rapid Library Prep (HT-RLP) kit (iGenomeX, San Diego, CA, USA). Whole genome sequencing was performed on the NovaSeq™ 6000 Sequencing System (Illumina Inc., San Diego, CA, USA) with the NovaSeq™ S Prime (SP) flow cell (Illumina Inc., San Diego, CA, USA) generating 2 × 150 bp paired-end reads (at an average of 190 × sequencing depth). Raw reads were trimmed using cutadapt (–nextseq-trim = 20) [19], Trimmomatic (leading 20, trailing 20, minimum length 25) [20] and ERNE-FILTER (–min-mean-phred-quality 30 –min-phred-value-mott 30 –sensitive) [21]. De novo assembly was performed using SPAdes 3.13.0 (–phred-offset 33 -k 35,55,75,95,115,127) [22] and assembly improvement was performed using Pilon 1.22 [23]. The assembled draft genomes/contigs were annotated using Prokka 1.14.6 [24] with default databases, as well as additionally installed TIGRFAMs (database version 2021–08-02) [25] and the Pfam hidden Markov model (HMM) databases (database version 2021–03-19) [26]. Multilocus sequence typing (MLST) was performed in silico using the mlst software (https://github.com/tseemann/mlst) based on the *P. aeruginosa* PubMLST database (https://pubmlst.org/organisms/pseudomonas-aeruginosa) [27]. The draft genomes were screened for antibiotic resistance genes (ARGs) and virulence factor genes (VFGs) in the Comprehensive Antibiotic Resistance Database (CARD) [28] and the VFDB [29] using ABRicate 1.0.1 (https://github.com/tseemann/abricate; database version 2021–09-22). Point mutations associated with fluoroquinolone resistance in the *gyrA*, *parC* and *parE* genes were detected by AMRFinder [30].

For determination of the genetic environment surrounding the *bla*_NDM-1_ gene, the annotated draft genomes were further investigated for the presence of flanking transfer RNA (tRNA) genes, mobility genes (transposases and integrases), insertion sequence (IS) elements and virulence genes with the following bioinformatics tools: Artemis [31], Artemis Comparison Tool (ACT) [32], BLASTN and BLASTP searches against the NCBI nucleotide (nt) and nr databases and the UniProt Knowledgebase [33], ICEfinder [34], ISfinder [35], IslandViewer 4 webserver (http://www.pathogenomics.sfu.ca/islandviewer/) [36] (ordered against *P. aeruginosa* isolate ST773, accession number NZ_CP041945.1; most closely related genome according to the PathoSystems Resource Integration Center (PATRIC) Similar Genome Finder [37]) and Mauve Contig Mover [38]. Easyfig version 2.2.2 [39] was used to visualise the linear comparison between (i) the putative ICE regions, ICE*6660-like* and ICE*6660*; and (ii) the *bla*_NDM-1_-surrounding regions in the study isolates and the corresponding regions in ICE*6660-like* and ICE*6660*.

### Phylogenetic analysis and calculation of the single nucleotide polymorphism difference matrix

For phylogenetic analysis, 19 complete and draft genome sequences of ST773 *P. aeruginosa* isolates (all available isolates that belonged to ST773 according the PATRIC website [37] as of 15 June 2021; 11 isolates were *bla*_NDM-1_-negative and eight were *bla*_NDM-1_-positive) were downloaded from the NCBI website (https://www.ncbi.nlm.nih.gov/genome/browse/#!/prokaryotes/187) (Table 1). Parsnp 1.5.6 from the Harvest Suite package [40] (with the “-x” option for filtering recombination sites based on PhiPack [41]) was used to obtain a recombination-free core genome by aligning draft genome sequences of the study isolates and the downloaded genome sequences (Table 1) against the reference genome (NZ_CP041945.1), of which prophage regions identified by PHASTER [42] were masked using the maskseq tool [43]. A recombination-free, core SNP-based maximum likelihood tree was constructed using RAxML 8.2.12 [44] with the rapid bootstrapping mode (“-f a”) and the GTRGAMMA model of nucleotide substitution, which was visualised using FigTree 1.4.4 (http://tree.bio.ed.ac.uk/software/figtree/). Branch support was estimated by 100 bootstrap replicates. The SNP difference matrix was calculated from the core genome alignment using the snp-dists 0.7.0 (https://github.com/tseemann/snp-dists). A previously suggested threshold of ≤ 37 SNP differences was used to define relatedness [45]. Hierarchical Bayesian clustering analysis was performed using fastbaps 1.0.6 [46] on the core SNP-only alignment in R 4.1.1 [47] (with the “optimise_baps” prior parameters and the “multi_res_baps” function for multi-level clustering). Clades were defined by using the first level of clustering and subclades by using the second level of clustering.

**Table 1:** List of the downloaded genome sequences of the ST773 isolates for the phylogenetic analysis from the NCBI genome database (https://www.ncbi.nlm.nih.gov/genome/browse/#!/prokaryotes/187)

Isolate	Specimen type^a^	Country^a^	*bla*_NDM-1_	Length^a^	Genes^a^	NCBI accession no
15,965	Urine	Nigeria	Positive	6,690,617	6263	GCA_017292115.1
PA790	Urine	India	Positive	6,932,250	6535	CP075176.1
PA-50010278	Surgical site	USA	Positive	6,821,270	6441	GCA_009791355.1
PS1	Urine	Hungary	Positive	6,720,818	6323	GCA_003725635.1
PSE6684	Urine	South Korea	Positive	6,924,367	6482	CP054917.1
ST773	Urine	USA	Positive	6,835,731	6400	CP041945.1
U1849	Unknown	Unknown	Positive	6,827,334	6446	GCA_003954525.1
U3484	Unknown	Unknown	Positive	6,870,810	6505	GCA_003954355.1
85	Surgical wound swab	Ghana	Negative	6,719,926	6346	GCA_002411915.1
60,503	Sputum	China	Negative	6,809,062	6385	CP041774.1
AZPAE14398	Intra-abdominal infection site	Germany	Negative	6,693,786	6323	GCA_000796095.1
AZPAE14889	Intra-abdominal infection site	China	Negative	6,479,466	6042	GCA_000790805.1
AZPAE14959	Intra-abdominal infection site	India	Negative	6,743,682	6337	GCA_000791735.1
NCTC13715	Urine	UK	Negative	6,765,311	6299	LR134330.1
PA_151908	Unknown	Hong Kong	Negative	6,879,776	6500	GCA_003585175.1
PMM38	Nasopharyngeal tissue	South Korea	Negative	6,662,434	6308	GCA_003836135.1
ZBX-P9	Urine	Lebanon	Negative	6,984,160	6568	GCA_017693745.1
ZBX-P14	Urine	Lebanon	Negative	7,375,847	6970	GCA_017693755.1
ZBX-P24	Endotracheal aspirate	Lebanon	Negative	7,107,128	6749	GCA_017693465.1

^a^Retrieved from the PATRIC website [37] and the *Pseudomonas* Genome Database [48] on 15 June 2021

## Results

### General features of the study isolates collected in the Gauteng region, South Africa

Three CRPA isolates that contained the *bla*_NDM-1_ gene were investigated in this study, of which the AST results are summarised in Table 2. All three isolates were resistant to all tested antibiotics, except for colistin (an XDR phenotype), and belonged to ST773 according to MLST. The genome size of the *bla*_NDM-1_-positive ST773 isolates varied between 7 and 7.1 Mbp and had similar guanine-cytosine (GC) content (65.61% to 65.78%) (Table 2).

**Table 2:** Characteristics of the *bla*_NDM-1_-positive carbapenem-resistant *P. aeruginosa* isolates investigated in the study

Isolate	Collection date	Specimen type	Hospital ward	City	VITEK MIC (μg·mL^−1^)								BMD MIC (μg·mL^−1^)	Genome size (bp)	Contigs	N50	GC content (%)
					TZP	CAZ	FEP	IPM	MEM	AMK	GEN	CIP	CST				
PA-A18	June 2019	Endotracheal aspirate (ETA)	High care	Boksburg	16 (R)	8 (R)	8 (R)	≥ 8 (R)	8 (R)	16 (R)	4 (R)	0.5 (R)	1 (S)	7,117,873	896	240,343	65.61
PA-D5^a^	March 2019	Pus	ICU	Benoni	≥ 128 (R)	≥ 64 (R)	≥ 32 (R)	U (R^a^)	≥ 16 (R)	32 (R)	≥ 16 (R)	≥ 4 (R)	0.5 (S)	7,017,822	621	240,904	65.77
PA-P104	December 2017	Sputum	General	Johannesburg	≥ 128 (R)	32 (R)	≥ 32 (R)	≥ 8 (R)	8 (R)	≥ 64 (R)	≥ 16 (R)	≥ 4 (R)	2 (S)	7,073,799	659	75,640	65.78

*AMK*, amikacin; *BMD*, broth microdilution; *CAZ*, ceftazidime; *CIP*, ciprofloxacin; *CST*, colistin; *FEP*, cefepime; *GEN*, gentamicin; *ICU*, intensive care unit; *IPM*, imipenem; *MEM*, meropenem; *MIC*, minimum inhibitory concentration; *R*, resistant; *S*, susceptible; *TZP*, piperacillin-tazobactam; *U*, unknown

^a^Resistance was confirmed by the disc diffusion assay

### Genetic relatedness, resistome and virulome of the study isolates in comparison with ST773 *P. aeruginosa* isolates from different countries

**Fig. 1:**
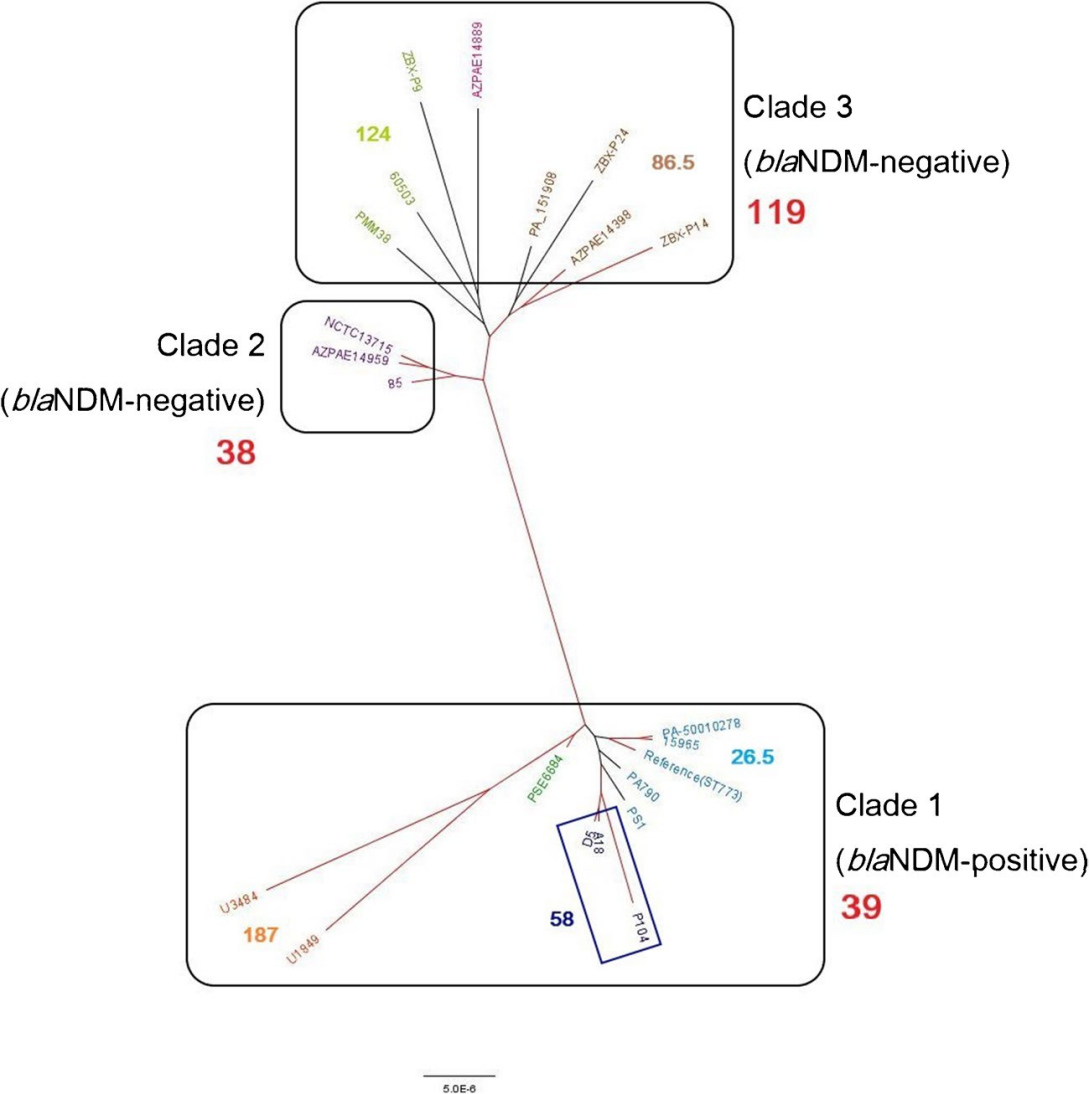
A recombination-free maximum likelihood tree based on the core SNP alignment against the reference genome (*P. aeruginosa* isolate ST773, CP041945.1). Three clades and eight subclades were identified by fastbaps 1.0.6 [46]. The bold number in red indicates the within-clade median SNP differences. Each colour represents subclades with bold number showing the within-subclade median SNP differences. The study isolates are indicated in blue box. Branches with bootstrap support ≥ 87% are coloured in red. Scale bar represents number of SNPs per variable site

Genetic relatedness of the study isolates in comparison with the 19 downloaded ST773 *P. aeruginosa* isolates from the NCBI website (referred as “ST773 isolates” below) is shown in Fig. 1. The pairwise core SNP differences between the study isolates and the 19 ST773 isolates within clades and subclades are listed in Table 3. The study isolates and the ST773 isolates had 192 median SNP differences (interquartile range (IQR), 93.5–231.5), which ranged from 13 to 393 SNP differences (Table 3). The hierarchical Bayesian clustering (fastbaps) analysis identified three clades (1st level of clustering), of which the study isolates and all eight *bla*_NDM-1_-positive ST773 isolates were grouped into the same clade (clade 1; 39 median SNP differences; IQR, 30.5–152) (Fig. 1; Table 3). Eleven *bla*_NDM-1_-negative ST773 isolates were grouped into clade 2 (38 median SNP differences; IQR, 30–40) and clade 3 (119 median SNP differences; IQR, 88–126.25), consisting of three and eight isolates, respectively (Fig. 1; Table 3). The 2nd level of clustering further grouped clade 1 into four subclades (subclades 1 to 4), clade 2 into subclade 5 and clade 3 into three subclades (subclades 6 to 8) (Fig. 1; Table 3). Although subclade 1 (the study isolates) and subclade 3 (five *bla*_NDM-1_-positive ST773 isolates (PA790, ST773, PS1, PA-50010278, 15965) from India, USA, Hungary and Nigeria) were separately grouped by fastbaps, these two subclades were closely related (34 median SNP differences; 39.4 average SNP differences; SNP difference range, 13–78; IQR, 28.5–39; Fig. 1). Amongst the study isolates, isolates PA-A18 and PA-D5 were highly related (13 SNP differences), whilst isolate PA-P104 was less related to the prior two isolates (59 and 58 SNP differences with isolates PA-A18 and PA-D5) (Fig. 1).

**Table 3:** The pairwise core SNP differences between the study isolates and 19 ST773 isolates within clades and subclades as identified by fastbaps

	Median	Average	Maximum	Minimum	IQR^a^
Clades (1st level of clustering)					
Clade 1 (PA-A18, PA-D5, PA-P104, PA790, ST773, PSE6684, PS1, PA-50010278, 15,965, U1849, U3484)	39	82.6	212	13	30.5–152
Clade 2 (NCTC13715, 85, AZPAE14959)	38	34	42	22	30–40
Clade 3 (PA_151908, PMM38, AZPAE14398, 60,503, AZPAE14889, ZBX-P9, ZBX-P14, ZBX-P24)	119	114.2	161	53	88–126.25
Inter-clade (clade 1 vs clade 2)	156	131.6	330	13	37–194.5
Inter-clade (clade 2 vs clade 3)	117	107.1	161	22	87–126
Inter-clade (clade 1 vs clade 3)	212	180.2	393	13	88–251
Subclades (2nd level of clustering)					
Subclade 1 (PA-A18, PA-D5, PA-P104)	58	43.3	59	13	35.5–58.5
Subclade 2 (U1849, U3484)	187	187	187	187	NC
Subclade 3 (PA790, ST773, PS1, PA-50010278, 15,965)	26.5	26	33	14	25–29
Subclade 4 (PSE6684)	0	0	0	0	NC
Subclade 5 (NCTC13715, 85, AZPAE14959)	38	34	42	22	30–40
Subclade 6 (PA_151908, AZPAE14398, ZBX-P14, ZBX-P24)	86.5	87	125	53	82–89.5
Subclade 7 (PMM38, 60,503, ZBX-P9)	124	111.7	125	86	105–124.5
Subclade 8 (AZPAE14889)	0	0	0	0	NC
Between isolates	192	177	393	13	93.5–231.5

^a^IQR was calculated for clusters containing three or more isolates. *IQR*, interquartile range; *NC*, not calculated; *SNP*, single nucleotide polymorphism; *ST*, sequence type

The study isolates harboured a wide variety of intrinsic and acquired ARGs that confer resistance to multiple antibiotics including aminoglycosides, β-lactam antibiotics (carbapenems, cephamycin, cephalosporins, monobactams), chloramphenicol, fluoroquinolones, fosfomycin, macrolides, sulfonamide, tetracycline and triclosan (Table 4). The intrinsic ARGs unique to all study isolates included *bla*_OXA-906_ (encodes an OXA-50 family β-lactamase; 99.37% (784/789 nucleotides) similar to *bla*_OXA-395_ found in the rest of ST773 isolates) and *bla*_PDC-19b_ (99.66% (1190/1194 nucleotides) similar to *bla*_PDC-385_ found in the rest of ST773 isolates). In addition, isolate PA-A18 contained the *dfr*B5 gene conferring trimethoprim resistance, which was absent in the other two isolates. The study isolates also contained acquired ARGs specific to clade 1, which included genes encoding the aminoglycoside-modifying enzymes (*aad*A11), carbapenemase (*bla*_NDM-1_), quinolone resistance pentapeptide repeat protein (*qnr*VC1) and the major facilitator superfamily (MFS)-type efflux pumps (*flo*R2/*cml*A9).

**Table 4:** Antibiotic resistance genes detected in the study isolates

Present in	Intrinsic resistance (chromosomal)			Acquired resistance		
	Resistance mechanisms	Antibiotic classes	Genes	Resistance mechanisms	Antibiotic classes	Genes
All	Antibiotic efflux (RND)	Bicyclomycin, carbapenem, cephalosporin, cephamycin, chloramphenicol, macrolide, fluoroquinolone, monobactam, sulfonamide, tetracycline, triclosan	*arm*R, *cpx*R, *mexAB-oprM*, *mexCD-oprJ*, *mexEF-oprN*, ***mexGHI-opmD*, *mexJK*, *mexL*,** *mexMN*, *mexPQ-opmE*, ***mexVW*, ***mexY*, *muxABC-opmB*, *opmH*, *soxR*, *triABC*, *yajC*	Antibiotic efflux (MFS)	Chloramphenicol	*cml*A9* (flo*R2*)*
				Antibiotic efflux (SMR)	Antiseptics (QAC)	*qac*EΔ1
				Antibiotic inactivation	Aminoglycoside	*aad*A11
					Fluoroquinolone	*crp*P, *qnr*VC1
	Antibiotic efflux (MFS)	Bicyclomycin, tetracycline	*bcr-1*, *tet*G		Carbapenems, cephamycin, cephalosporin	*bla*_NDM-1_
	Antibiotic efflux (MATE)	Antiseptics, aminoglycoside, fluoroquinolone	*pmp*M	Antibiotic target modification	Aminoglycoside	*rmt*B4
	Antibiotic efflux (SMR)	Aminoglycoside	*emr*E	Antibiotic target substitution	Sulfonamide	*sul*1
	Antibiotic inactivation	Aminoglycoside	*aac*C3^a^, *aph*(3ʹ)-IIb			
		Carbapenems, cephamycin, cephalosporin	***bla***_**OXA-906**_**, *bla***_**PDC-19b**_			
		Chloramphenicol	*cat*B7			
		Fosfomycin	*fos*A			
	Antibiotic target modification	Fluoroquinolone	*gyr*A^b^ (T83I mutation), *par*C^b^ (S87L mutation)			
PA-A18 and PA-D5 only	Antibiotic efflux (RND)	Bicyclomycin, carbapenem, cephalosporin, cephamycin, chloramphenicol, macrolide, fluoroquinolone, monobactam, sulfonamide, tetracycline, triclosan	*mex*X			
PA-A18 only				Antibiotic target substitution	Sulfonamide	***dfr*B5**

Genes in bold indicate unique genes that were present in the study isolates only; underlined genes indicate genes specific to clade 1. *MATE*, multidrug and toxic compound extrusion; *MFS*, major facilitator superfamily; *QAC*, quaternary ammonium compound; *RND*, resistance-nodulation-cell division; *SMR*, small multidrug resistance

^a^Manually identified from the Prokka annotations and BLASTN searches against the NCBI nucleotide (nt) database [49]

^b^Detected by AMRFinder [30]

The search against the virulence factor database (VFDB) revealed that the study isolates harbour 12 different types of VFGs, which included genes responsible for alginate production, the *las* and *rhl* quorum sensing systems, and pyochelin and pyoverdine production (iron uptake), as well as genes encoding type IV pili, flagella, lipopolysaccharide (LPS) and protein secretion systems (type II, III, IV and VI) (Table 5). The VFGs unique to the study isolates were genes encoding the VI secretion systems (*hsi*B1/*vip*A, *hsi*C1/*vip*B, *hsi*F1, *hsi*G1, *hsi*H1).

**Table 5:** Virulence factor genes detected in the study isolates (based on VFDB)

Virulence factor	Genes
Alginate production (immune evasion)	*alg*44, *alg*8, *alg*A, *alg*B, *alg*C, *alg*D, *alg*E, *alg*F, *alg*G, *alg*I, *alg*J, *alg*K, *alg*L, algP/algR3^c^, *alg*Q, *alg*R, *alg*U, *alg*W, *alg*X, *alg*Z, *muc*A, *muc*B, *muc*C, *muc*D, *muc*E, *muc*P
Flagella and type IV pili (attachment)	*chp*A, *chp*B, *chp*C, *chp*D, *chp*E, ***fim*G**^**a**^, *fim*V, *fle*N, *fle*Q, *fle*R, *fle*S, *flg*A, *flg*B, *flg*C, *flg*D, *flg*E, *flg*F, *flg*G, *flg*H, *flg*I, *flg*J, *flg*K, *flg*M, *flg*N, *flh*A, *flh*B, *flh*F, *fli*A, *fli*E, *fli*F, *fli*G, *fli*H, *fli*I, *fli*J, *fli*K, *fli*L, *fli*M, *fli*N, *fli*O, *fli*P, *fli*Q, *fli*R, *mot*A, *mot*B, *mot*C, *mot*D, *mot*Y, *pil*B, *pil*F, *pil*G, *pil*H, *pil*I, *pil*J, *pil*K, *pil*M, *pil*N, *pil*O, *pil*P, *pil*Q, *pil*R, *pil*S, *pil*T, *pil*U
Iron uptake (pyochelin and pyoverdine production)	*fpt*A, *mbt*H-like, *pch*A, *pch*B, *pch*C, *pch*D, *pch*E, *pch*F, *pch*G, *pch*H, *pch*I, *pch*R, *pvc*A, *pvc*B, *pvc*C, *pvc*D, *pvd*A, *pvd*F, *pvd*G, *pvd*H, *pvd*L^a^, *pvd*M, *pvd*N, *pvd*O, *pvd*P, *pvd*Q, *pvd*S
Lipopolysaccharide	*waa*A, *waa*C, *waa*F, *waa*G, *waa*P
Phospholipase C	*plc*H
Protease and elastase	*apr*A, *las*A, *las*B
Quorum sensing	*las*I, *rhl*A, *rhl*B, *rhl*C, *rhl*I
Toxin production (pyocyanin, exotoxin A)	*phz*A1, *phz*B1, *phz*D1, *phz*E1, *phz*F1, *phz*G1, *phz*H, *phz*M, *phz*S, *ptx*R *tox*A,
Type II secretion system	***gsp*M**^**b**^*, xcp*P, *xcp*Q, *xcp*R, *xcp*S, *xcp*T, *xcp*U, *xcp*V, *xcp*X, *xcp*Y, *xcp*Z
Type III secretion system	*exo*T, *exo*U, *exo*Y, *ptx*R, *exs*A, *exs*B, *exs*C, *exs*D, *exs*E, *pcr*1, *pcr*2, *pcr*3, *pcr*4, *pcr*D, *pcr*G, *pcr*H, *pcr*R, *pcr*V, *pop*B, *pop*D, *pop*N, *psc*B, *psc*C, *psc*D, *psc*E, *psc*F, *psc*G, *psc*H, *psc*I, *psc*J, *psc*K, *psc*L, *psc*N, *psc*O, *psc*P, *psc*Q, *psc*R, *psc*S, *psc*T, *psc*U, ***spc*U**
Type IV secretion system	*tse*1, *tse*2, *tse*3
Type VI secretion system	*clp*V1, *dot*U1, *fha*1, *hcp*1, *hsi*A1**, *hsi*B1/*vip*A, *hsi*C1/*vip*B**, *hsi*E1/*tag*J1, ***hsi*F1, *hsi*G1, *hsi*H1,** *hsi*J1, *icm*F1/*tss*M1, *lip*1, *ppk*A, *ppp*A, *tag*F/*ppp*B, *tag*Q, *tag*R*, tag*S*, tag*T, *vgr*G1a, *vgr*G1b

Genes in bold indicate unique genes that were present in the study isolates only

^a^Present in isolate PA-D5 only

^b^Present in isolate PA-A18 only

### Genetic context of the *bla*_NDM-1_ gene

**Fig. 2:**
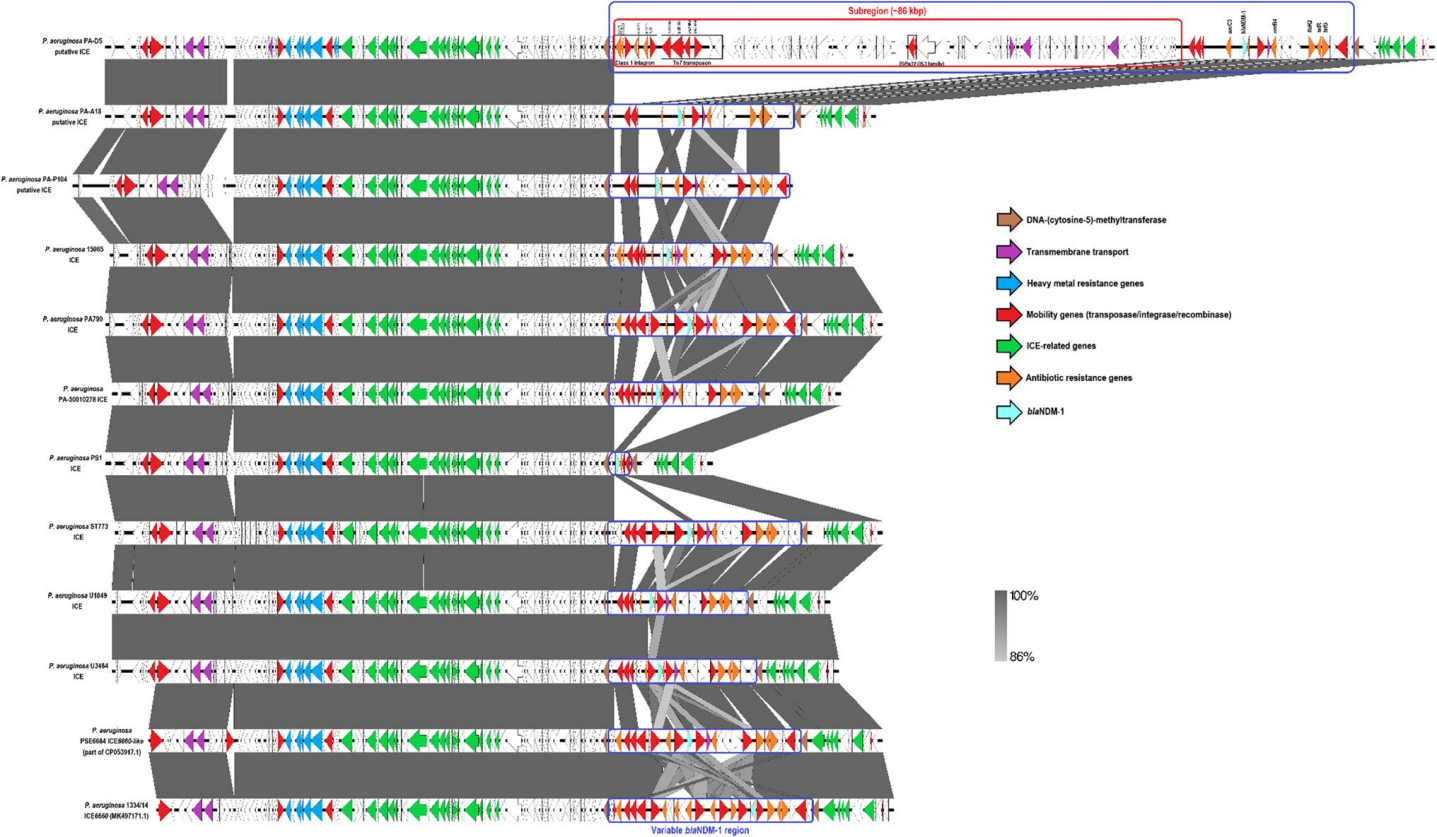
Graphical representation of putative integrative and conjugative elements (ICEs) found in study isolates and seven *bla*_NDM-1_-positive ST773 isolates, in comparison with ICE*6660-like* (part of CP053917.1) and ICE*6660* (MK497171.1). The image was generated by Easyfig version 2.2.2 [39]. The ICE*6660-like* and the ICE*6660* were reversed for better comparison. Arrows indicate all genes/coding sequences (CDSs) proportional to its size and orientation. Each colour of the arrow represents type of genes as indicated in the legend. Δ indicates a truncated/incomplete gene. Grey shades show homologous regions by nucleotide BLAST comparison and the sequence identity level

The characteristics of the putative *bla*_NDM-1_-carrying ICEs found in isolates PA-D5, PA-A18 and PA-P104 and seven *bla*_NDM-1_-positive ST773 isolates in comparison with the ICE*6660* (MK497171.1) from isolate “1334/14” (CP035739.1) and the ICE*6660-like* from isolate PSE6684 (CP053917.1) are summarised in Table 6. The *bla*_NDM-1_ gene was found in the putative integrative and conjugative element (ICE) regions varying in sizes from ~117 to ~204 kilobase pairs (kbp), which had the same 23-bp direct repeat sequence (5ʹ-GTCTCGTTTCCCGCTCCAAACAT-3ʹ) at both terminal ends and were integrated within the tRNA^Gly^ gene (Table 6). The “variable *bla*_NDM-1_ region” (size 113,940 bp (PA-D5); 28,224 bp (PA-A18); 27,773 bp (PA-P104)), with almost identical orientation and structure except for isolates PA-D5 and PS1, was always found inserted within the DNA methyltransferase gene in all study isolates and eight ST773 isolates, as well as in ICE*6660-like* (29,492 bp) and ICE*6660* (30,933 bp) (Fig. 2). Most notably, a unique “subregion” (85,907 bp) was found within the “variable *bla*_NDM-1_ region” in isolate PA-D5, which was absent in the other two study isolates (PA-A18 and PA-P104), eight ST773 isolates, ICE*6660* and ICE*6660-like*. This “subregion” was completely identical (100% coverage; 100% identity) to a genomic region in the *P. aeruginosa* PSE6684 genome (position 2,815,086 to 2,900,992), which included a putative class 1 integron (containing the plasmid-mediated “quinolone resistance determinant” VC1 (*qnr*VC1) and an aminoglycoside-modifying enzyme (*aad*A11) gene), a Tn*7* transposon and the IS*Pa32* (IS*3* family) element.

**Table 6:** Characteristics of the *bla*_NDM-1_-carrying ICEs in comparison with ICE*6660* (MK497171.1) and ICE*6660-like* (part of CP053917.1)

	PA-A18	PA-D5	PA-P104	15,965	PA790	PA-50010278	PS1	ST773	U1849	U3484	ICE*6660-like* (from PSE6684)	ICE*6660* (from 1334/14)
Size (bp)	117,235	203,624	120,661	113,145	118,240	110,843	92,342	116,839	109,291	110,601	111,700	112,205
GC content (%)	62.61	62.09	62.5	62.73	62.94	62.7	63.03	62.9	62.61	62.68	63.83	63.87
Insertion site	tRNA^Gly^	tRNA^Gly^	tRNA^Gly^	tRNA^Gly^	tRNA^Gly^	tRNA^Gly^	tRNA^Gly^	tRNA^Gly^	tRNA^Gly^	tRNA^Gly^	tRNA^Gly^	tRNA^Gly^
Direct repeat sequences (*att* site)	5ʹ-GTCTCGTTTCCCGCTCCAAACAT-3ʹ	5ʹ-GTCTCGTTTCCCGCTCCAAACAT-3ʹ	5ʹ- GTCTCGTTTCCCGCTCCAAACAT-3ʹ	5ʹ- GTCTCGTTTCCCGCTCCAAACAT-3ʹ	5ʹ- GTCTCGTTTCCCGCTCCAAACAT-3ʹ	5ʹ- GTCTCGTTTCCCGCTCCAAACAT-3ʹ	5ʹ- GTCTCGTTTCCCGCTCCAAACAT-3ʹ	5ʹ- GTCTCGTTTCCCGCTCCAAACAT-3ʹ	5ʹ- GTCTCGTTTCCCGCTCCAAACAT-3ʹ	5ʹ- GTCTCGTTTCCCGCTCCAAACAT-3ʹ	5ʹ-TTGGAGCGGGAAACGAGAC-3ʹ	5ʹ-TTGGAGCGGGAAACGAGAC-3ʹ
ARGs	*aac*C3, *bla*_NDM-1_, Δ*ble*_MBL_, *rmt*B4, *flo*R, *qac*EΔ1, *qnr*VC1, Δ*sul*1, *tet*R, *tet*G	*aac*C3, *bla*_NDM-1_, Δ*ble*_MBL_, *rmt*B4, *flo*R, *tet*R, *tet*G	*aac*C3, *bla*_NDM-1_, Δ*ble*_MBL_, *rmt*B4, *flo*R, *tet*R, *tet*G	*aac*C3, *bla*_NDM-1_, Δ*ble*_MBL_, *rmt*B4, *flo*R, *qac*EΔ1, *sul*1, *tet*R, *tet*G	*aac*C3, *bla*_NDM-1_, Δ*ble*_MBL_, *rmt*B4, *flo*R, *qac*EΔ1, *sul*1, *tet*R, *tet*G	*aac*C3, *bla*_NDM-1_, Δ*ble*_MBL_, *rmt*B4, *flo*R, *qac*EΔ1, *tet*R, *tet*G	*bla*_NDM-1_, Δ*ble*_MBL_	*aac*C3, *bla*_NDM-1_, Δ*ble*_MBL_, *rmt*B4, *flo*R, *qac*EΔ1, *tet*R, *tet*G	*aac*C3, *bla*_NDM-1_, Δ*ble*_MBL_, *rmt*B4, *flo*R, *qac*EΔ1, *tet*R, *tet*G	*aac*C3, *bla*_NDM-1_, Δ*ble*_MBL_, *rmt*B4, *flo*R, *qac*EΔ1, *tet*R, *tet*G	*aac*C3, *bla*_NDM-1_, Δ*ble*_MBL_, *flo*R2, *qac*EΔ1, *rmt*B4, *sul*1, *tet*R, *tet*G	*aac*C3, *bla*_NDM-1_, *bla*_PME-1_, Δ*ble*_MBL_, *flo*R, *rmt*D3, *sul*1, *tet*R, *tet*G
BLASTN similarity with ICE*6660*	Sequence identity, 99.99% Coverage, 82%	Sequence identity, 99.99% Coverage, 48%	Sequence identity, 99.99% Coverage, 80%	Sequence identity, 99.99% Coverage, 93%	Sequence identity, 99.81% Coverage, 94%	Sequence identity, 99.99% Coverage, 92%	Sequence identity, 99.99% Coverage, 75%	Sequence identity, 99.99% Coverage, 94%	Sequence identity, 99.99% Coverage, 91%	Sequence identity, 99.99% Coverage, 91%	N/A	N/A
BLASTN similarity with ICE*6660-like*	Sequence identity, 100% Coverage, 87%	Sequence identity, 100% Coverage, 51%	Sequence identity, 86% Coverage, 100%	Sequence identity, 99.99% Coverage, 97%	Sequence identity, 99.99% Coverage, 100%	Sequence identity, 99.99% Coverage, 96%	Sequence identity, 100% Coverage, 77%	Sequence identity, 99.98% Coverage, 98%	Sequence identity, 100% Coverage, 96%	Sequence identity, 100% Coverage, 96%	N/A	N/A

*ARGs*, antibiotic resistance genes; *bp*, base pairs; *ICE*, integrative and conjugative element; *N/A*, not applicable

**Fig. 3:**
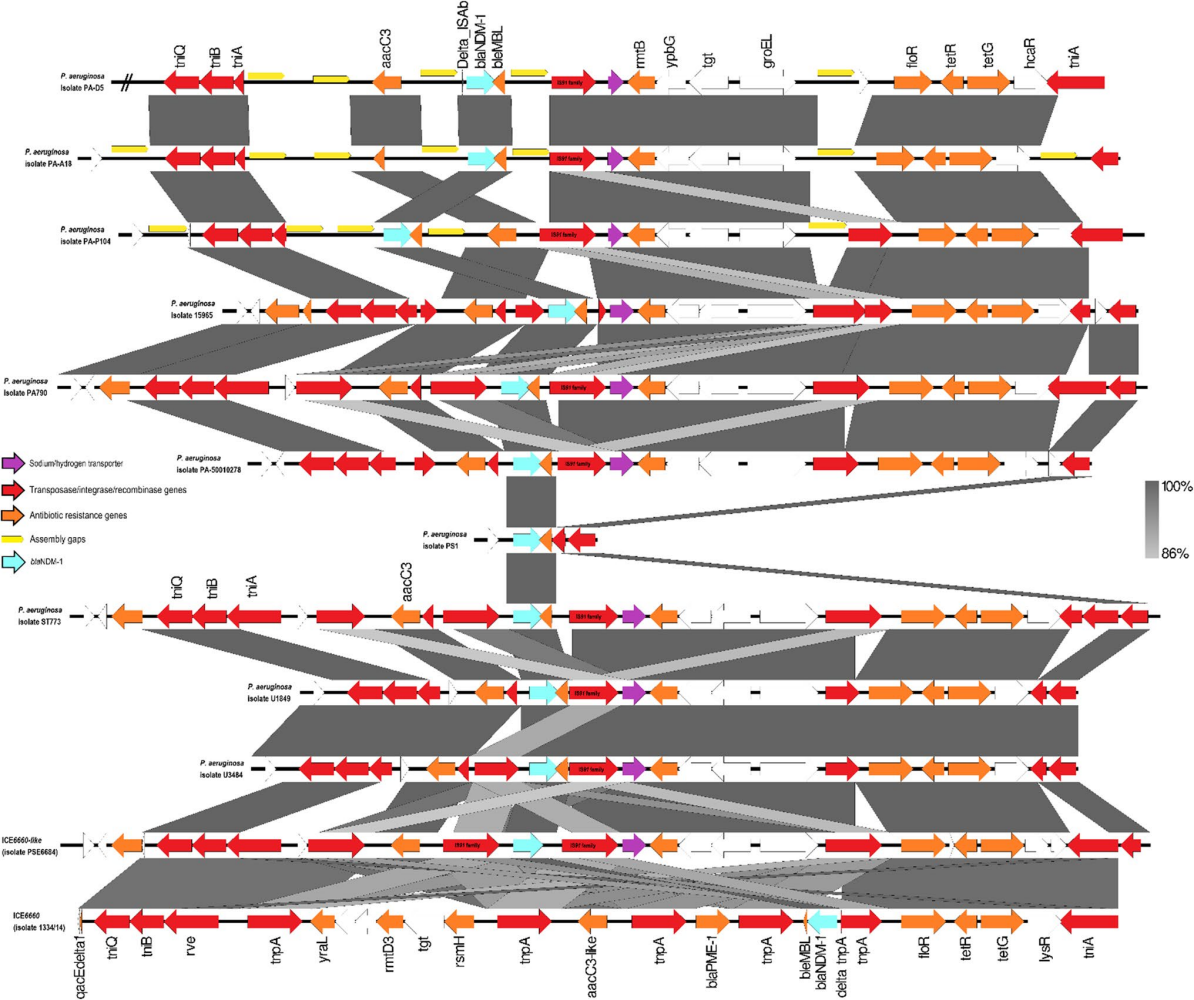
Comparison of the “variable *bla*_NDM-1_ region” of study isolates, seven *bla*_NDM-1_-positive ST773 isolates, ICE*6660-like* (part of CP053917.1) and ICE*6660* (MK497171.1). The ICE*6660-like* and the ICE*6660 bla*_NDM-1_ regions were reversed and the isolate PA-D5 subregion was omitted for better comparison. Image was generated using Easyfig 2.2.2 [39]. Arrows indicate all genes/coding sequences (CDSs) with each colour representing each type of genes and yellow frames indicate assembly gaps. Grey shades indicate homologous regions by nucleotide BLAST comparison and the sequence identity levels. Δ indicates a truncated gene

Likewise, the genetic environment in the “variable *bla*_NDM-1_ region” was almost similar for two study isolates (PA-D5 and PA-A18), six ST773 isolates (15,965, PA790, PA-50010278, ST773, U1849 and U3484) and ICE*6660-like* (Fig. 3). The IS*91* family transposase gene was almost always found downstream of the ΔIS*Aba125*-*bla*_NDM-1_-Δ*ble*_MBL_ gene set, with only exception that the aminoglycoside-(3)-N-acetyltransferase III gene (*aacC3*) was located downstream of the ΔIS*Aba125*-*bla*_NDM-1_-Δ*ble*_MBL_ gene set in isolate PA-P104 and that it was absent in isolate PS1 (Fig. 3). The ARGs specific to clade 1 (*flo*R2/*cml*A9, *rmt*B4, *tet*G) other than the *bla*_NDM-1_ and Δ*ble*_MBL_ genes were always found within the “variable *bla*_NDM-1_ region” except for isolate PS1.

## Discussion

The emergence and worldwide dissemination of CRPA and international high-risk clones have become a major public health threat due to its ability to acquire genes producing carbapenemases, aminoglycoside-modifying enzymes and quinolone resistance determinants through HGT of MGEs such as integrons and ICEs [5, 6]. The present study describes the genomic and molecular epidemiological characteristics of three *bla*_NDM-1_-positive CRPA isolates detected in the Gauteng region, South Africa. The key findings of the study were as follows: (i) the study isolates belonged to the same clade with the *bla*_NDM-1_-positive global ST773 isolates, which shared a few clade-specific ARGs/molecular markers; (ii) the study isolates showed an XDR phenotype and had a wide range of intrinsic and acquired ARGs (including ARGs related with MGEs, porins and efflux pumps) and VFGs; and (iii) the clade-specific ARGs (*bla*_NDM-1_, *flo*R2/*cml*A9, *rmt*B4, *tet*G) were found in the similar genetic environment as in the ICE*6660-like*.

ST773 is an international high-risk clone associated with MDR and XDR phenotypes that produce MBLs (VIMs, IMPs, NDMs), which is widely distributed in countries like Hungary [50], India [51], Nepal [52], Nigeria [53], Saudi Arabia [54], South Korea [55], the UK [56] and the USA [57]. The core SNP-based phylogenetic analysis of the study isolates with 19 global ST773 isolates revealed that the study isolates and the eight *bla*_NDM-1_-positive ST773 isolates belonged to the same clade. Especially, the CRPA isolates were closely related (median SNP differences, 34; IQR 28.5–39; Fig. 1) to five *bla*_NDM-1_-positive ST773 isolates (PA790, ST773, PS1, PA-50010278, 15,965) from India, the USA, Hungary and Nigeria, which may indicate a recently shared origin of the study isolates with isolates from these countries. Previous hospitalisation or international travel in endemic countries such as India is an important risk factor for colonisation or infection of carbapenemase-producing, MDR or XDR Gram-negative bacteria (including NDM-producing *P. aeruginosa* and *Enterobacterales*) in returning countries (39–42). However, further investigation is required to confirm this finding as no travel history of patients was available in this study.

In the study isolates and seven *bla*_NDM-1_-positive ST773 isolates (clade 1), the *bla*_NDM-1_ gene was found within the ~117- to ~204-kbp putative ICE regions similar to the ICE*6660-like*. Interestingly, the majority of the clade-specific ARGs (*flo*R2/*cml*A9, *rmt*B4, *tet*G) were found in proximity with *bla*_NDM-1_ (except for isolate PS1), whilst the class 1 integrons containing *aad*A11 and *qnr*VC1 were also found within the “variable *bla*_NDM-1_ region” in isolate PA-D5. These findings may suggest that this particular *bla*_NDM-1_-positive ST773 clade may have emerged by acquiring ICEs and integrons carrying ARGs that confer resistance to aminoglycosides, carbapenems, chloramphenicol, fluoroquinolones and tetracyclines. This is consistent with previous studies that suggest the *bla*_NDM-1_ and *qnr*VC1 genes as molecular markers of ST773 and that there is some phylogenomic preference in the *bla*_NDM-1_ gene acquisition in *P. aeruginosa* [50, 55, 58]. The ICE specifically carrying the *aac*C3, *bla*_NDM-1_ and *rmt*B4 genes was first reported in the *P. aeruginosa* isolate “ST773” in the USA in 2019, which was obtained from a returning traveller that underwent surgery in India [59]. The ICE with the same genetic structure, composition and direct repeat sequences, designated as ICE*6660-like*, was also recently found in South Korea [55], Nepal [52], Saudi Arabia [54] and Nigeria [53]. The international distribution of the *bla*_NDM-1_-positive ST773 clade carrying ARGs on the genetic platforms such as ICEs warrants special attention since ICEs are self-transmissible and can facilitate intra- and intercellular mobility of ARGs [60, 61] to other carbapenem-susceptible *P. aeruginosa* isolates and other Gram-negative bacteria (*Enterobacterales*) in the same region and other countries by travelling, which may further increase the health burden caused by MDR and XDR *P. aeruginosa* infections.

Other than ARGs, the WGS analysis of the ST773 CRPA isolates in this study revealed a wide repertoire of cell-associated (flagella, type IV pili, alginate, LPS, type II, III, IV and VI protein secretion systems) and extracellular VFGs (proteases, phospholipase C, pyoverdine, pyochelin, pyocyanin, exotoxin A). These findings are not surprising as ST773 has often been associated with many VFGs such as *apr*A, *alg*D, *exo*T, *exo*U, *exo*Y, *las*A, *las*B, *phz*M, *phz*S and *tox*A [62, 63]. Although it is known that virulence of *P. aeruginosa* may be suppressed whilst being MDR or XDR as a result of the fitness cost, the *exo*U^+^-genotype STs such as ST235 have been found to be highly virulent and were associated with early mortality in patients with BSIs [64–66]. The presence of the *exo*U gene and a wide variety of VFGs in the study isolates may show the virulence potential of the *bla*_NDM-1_-positive ST773 clade, which may further pose a challenge for the recovery of critically ill patients in the hospitals.

To the authors’ knowledge, this is the first study to report genomic characteristics of *bla*_NDM-1_-positive ST773 CRPA isolates in South Africa and the genetic environment of the *bla*_NDM-1_ gene in these CRPA isolates. However, the authors acknowledge some limitations. First, BMD was performed to CST only and novel β-lactam combinations such as ceftolozane/tazobactam, ceftazidime/avibactam and cefiderocol were not tested. In South Africa, these antibiotics are not available in public sectors and are often not available due to stock issues [67]. Second, no epidemiological information (e.g. travel history) was available in this study to confirm any direct epidemiological link of the ST773 isolates. Third, draft genomes and putative ICE predictions in this study were obtained by short read sequencing only, which resulted in fragmented contigs with some assembly gaps. In future, a hybrid assembly approach using both short- and a long-read sequencing technologies such as Oxford Nanopore DNA sequencing (Oxford Nanopore Technologies, Oxford, UK) or PacBio single molecule real-time (SMRT) sequencing (PacBio®, Menlo Park, CA, USA) could be useful in obtaining complete genome sequences and sequences of longer MGEs such as ICEs and plasmids.

In conclusion, this study reports the presence of the *bla*_NDM-1_-positive ST773 clone in the Gauteng region, South Africa, and the carriage of the *bla*_NDM-1_ gene and ARGs on a putative ICE similar to ICE*6660-like*. Continuous and active molecular/genomic surveillance of CRPA in the Gauteng region, South Africa, is needed to monitor the emergence and spread of clones harbouring the carbapenemase gene and VFGs to prevent its establishment and transmission in healthcare settings.

## Data Availability

The draft genome sequences of the isolates PA-A18, PA-D5 and PA-P104 have been deposited to the public database (NCBI GenBank database) with the following accession numbers: JAVKRX000000000, JAVKRY000000000, JAVKRZ000000000, under the BioProject no. PRJNA1010667. Supporting data and protocols have been provided within the article.
